# Development and Characterization of 20 Genomic SSR Markers for Ornamental Cultivars of *Weigela*

**DOI:** 10.3390/plants11111444

**Published:** 2022-05-29

**Authors:** Trinity P. Hamm, Sarah L. Boggess, Jinita Sthapit Kandel, Margaret E. Staton, Matthew L. Huff, Denita Hadziabdic, DeWayne Shoemaker, John J. Adamczyk Jr., Marcin Nowicki, Robert N. Trigiano

**Affiliations:** 1 Department of Entomology and Plant Pathology, University of Tennessee, Knoxville, TN 37996, USA; mstaton1@utk.edu (M.E.S.); mhuff10@utk.edu (M.L.H.); dhadziab@utk.edu (D.H.); dewayne.shoemaker@utk.edu (D.S.); mnowicki@utk.edu (M.N.); 2 Thad Cochran Southern Horticultural Research Laboratory, USDA ARS, Poplarville, MS 39470, USA; jinita.sthapit@usda.gov (J.S.K.); john.adamczyk@usda.gov (J.J.A.J.)

**Keywords:** Caprifoliaceae, genetic diversity, gSSR, microsatellites

## Abstract

*Weigela* (Caprifoliaceae) is a genus of ornamental plants popular for its phenotypic variation and hardiness, that includes species hybridized to produce the commercially available cultivars. Despite its popularity, limited genetic resources exist for the genus. Twenty genomic simple sequence repeat (gSSR) markers distributed across the genome were developed using low coverage whole-genome sequencing data of *Weigela* Spilled Wine^®^. A cross-amplification evaluation with these 20 gSSR markers on a collection of 18 *Weigela* cultivars revealed a total of 111 unique alleles, including 36 private alleles. A diagrammatic key was constructed to identify cultivars using only six of the gSSR markers, demonstrating the newly developed gSSR markers are immediately useful for cultivar identification. Future uses could include breeding with marker-assisted selection, determining the history of hybridization of the current cultivated lines, aiding in the construction of genetic maps, and assessing the patterns of population genetic structure of *Weigela* spp.

## 1. Introduction

*Weigela* Thunb., classified within the Caprifoliaceae (honeysuckle family), is a small genus comprised of roughly 10–12 species, all native to eastern Asia [1,2,3]. *Weigela* plants are deciduous, hardy shrubs that bloom with five-lobed, trumpet-shaped flowers and can grow up to 5 m. They grow well in full sun with moist, but well-drained soil, and are easily propagated with softwood cuttings in summer and hardwood cuttings in fall and winter [4]. Flowers appear in the spring months and their colors include white, pink, crimson, red, and yellow [2,5]. The re-blooming cultivars provide additional flowers throughout the summer. Growth habit and leaf color/variegation also vary across the cultivars. *Weigela* are popular in temperate ornamental landscapes, primarily in USDA Plant Hardiness zones four to eight. In 2019 alone, the U.S. market value of all sales of *Weigela* was USD14.26 million [6]. 

*Weigela* species are native to Northeast Asia with the highest known species diversity found in Japan and Korea [1,5]. The genus was introduced to Europe in the mid-nineteenth century, and European nurseries quickly started breeding programs to produce new hybrids [5]. A “check-list” of *Weigela* cultivar names was published by The Arnold Arboretum of Harvard University, Boston, Massachusetts, U.S., that listed cultivar names from nursery catalogs, horticultural magazines, botanical gardens, and arboreta in the U.S. and Europe [7]. In this publication, Howard recognized the confusion and inconsistencies that existed in the taxonomical treatment of this genus and its hybrid cultivars as early as 1965. Many cultivars are products of multiple hybridization events among *Weigela* spp., making classification with traditional systems difficult [1]. A new approach was proposed based solely on growers’ practices and phenotypic characterization, including plant size, flower color, and leaf color. *Weigela* cultivars were classified with this system into the following eight groups: Purpurea, Dwarf, Variegata, Aurea, White-flowered, Red-flowered, Pink-flowered, and Bicolor [1]. Further breeding history is largely unknown.

*Weigela* is a popular ornamental genus for its flower and leaf colors, but our knowledge regarding plant genetics and genetic diversity is very limited. *Weigela* spp. were previously classified within genus *Diervilla* Mill., and the remaining *Diervilla* species are all native to North America [2,3]. Nucleotide sequence variation in the internal transcribed spacer (ITS) regions of 18–26 S nuclear ribosomal DNA was used to reconstruct phylogenetic relationships among eleven species of *Weigela* and three species of *Diervilla* [3]. The resulting phylogenetic trees did not support the monophyly of *Weigela*, which was broken into three clades. Most *Weigela* spp. consistently fell into one well-supported clade, with the exception of two species. The relationship of *Weigela maximowiczii* was equivocal to the rest of the *Weigela* species. *Weigela middendorffiana* was the only *Weigela* species reliably found to be sister to the *Diervilla* clade. Intergeneric hybrids between *Weigela* and *Diervilla* have been developed using biotechnology tools, including ovule culture, micropropagation, and molecular screening techniques. However, the developed hybrids had low vigor and poor growth under both greenhouse and field conditions [8].

Relative genome size and ploidy level studies of different species and cultivars of *Weigela* suggest diploid genome sizes range from 1.91 to 2.32 picograms of DNA [2*n* = 2x = 36; 4]. There was only one triploid (2*n* = 3*x* = 54) cultivar, *W*. ‘Courtalor’ Carnaval^®^, among the 46 cultivars screened. *Weigela* ‘Courtalor’ Carnaval^®^ was one of the three triploid selections developed from colchicine treatment by Duron and Decourtye [2]. *Weigela florida* was included in a study comparing chloroplast sequence variation across the order Dipsacales to elucidate phylogenetic relationships among the species [9]. The study placed *W*. *florida* in the Diervilleae clade, which was the earliest diverging lineage in the Caprifoliaceae. Simple sequence repeat markers (SSRs, or microsatellites) were developed for *W. coraeensis* [10] and optimized for a variety endemic to the Japanese Izu Islands [11], although this is not a popular species used in breeding programs [1]. Developing additional genetic markers for *Weigela* cultivars would provide more genetic resources for assessing population structure in the wild, as well as exploring the genetic diversity of germplasm to guide future breeding efforts of the genus.

SSRs are sequences with a variable number of repeats of a few nucleotides, usually two to five [12]. SSRs are distributed ubiquitously throughout the genomes in eukaryotes, and when exploited as markers, are among the most informative molecular markers for population genetic studies due to their high mutation rate [13,14,15]. The variability in the number of repeats in SSRs is most likely caused by slippage during replication or DNA repair [16,17]. SSR markers are highly polymorphic, found abundantly across the genome, characteristically codominant, and readily amenable to automation for detection. These markers will also often cross-amplify in other closely related species [18]. SSRs can be mined and developed from genomic (gSSRs) or expressed sequence tag (e- or EST-SSRs) sequences, each with their own advantages and disadvantages [19,20]. SSR markers in general have been widely used in genetic studies including genetic diversity and population structure [21,22,23], constructing genetic maps [24,25], and quantitative trait loci (QTL) mapping [26,27]. Additionally, SSR markers can be used as a quick, low cost tool to identify cultivars, which is useful in breeding programs [28].

The objectives of this study were to (1) develop SSR markers from *Weigela* Spilled Wine^®^; (2) test the ability of the developed markers to amplify sequences in various cultivars despite their complicated breeding history; and (3) estimate genetic diversity across a subset of currently available cultivars from different breeding programs. The SSR markers developed in this study can potentially be used in the broader germplasm for identification of *Weigela* and closely related taxa, in future population genetic studies, and in breeding programs for cultivar identification and/or marker-assisted selection.

## 2. Results and Discussion

### 2.1. gSSR Marker Development

**Figure 1:**
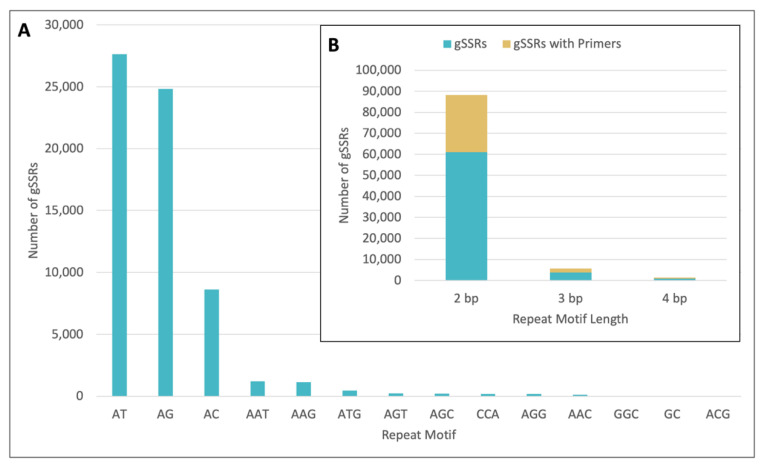
Genomic SSRs (gSSRs) mined from the de novo assembled genome of *Weigela* Spilled Wine^®^. Overall number of 2 bp and 3 bp gSSRs identified are shown in blue broken down by repeat motif (**A**). Distribution of 2 bp, 3 bp, and 4 bp repeat motif lengths for gSSRs (blue) and gSSRs with primers (gold) (**B**). bp = base pairs.

Paired-end whole-genome sequencing resulted in 14,029,819 raw reads, which represents approximately 4X genome coverage, based on an estimated 2C genome content of 2.06 pg for Spilled Wine^®^ [4]. The reads were assembled to yield 4,164,207 sequences spanning 833,684,734 bp. A total of 70,913 gSSRs were identified from 65,634 sequences, including 5156 classified as compound gSSRs (i.e., gSSRs next to each other or separated by less than 15 base pairs [bp]). Similar to other studies, the most common motif was [AT]_n_ [22,23,29], with 27,618 gSSRs or 45% of the 61,075 dinucleotide repeats being identified as [AT]_n_ (Figure 1). Additionally, 3761 tri- and 921 tetra-nucleotide repeats were identified (Figure 1). Primers were designed for 29,625 gSSRs, and 50 were selected for testing. Of the 50 randomly selected gSSR markers that were tested, 20 had no spurious banding and less than 30% missing data. The resulting 20 gSSR markers were used to genotype 18 *Weigela* cultivars with samples from two independent plants for 16 of the cultivars and one plant for two of the cultivars for a total of 34 entries. 

### 2.2. gSSR Characteristics

The 20 gSSR markers identified 111 alleles in the 18-cultivar *Weigela* collection and ranged from two to eight alleles per locus with a mean of 5.6 alleles per locus (Table 1). The full allele size dataset can be found in supplementary information (Table S1). Individual samples and loci were discarded from the study if they had greater than 30% missing data. Wei002, Wei035, and Wei044, had up to 29% missing data but were kept in downstream analyses. PCR amplifications at these loci failed after three attempts on certain cultivars, but were kept due to robust amplification in the other cultivars tested. Wei002 had robust amplification in 15/18 cultivars; Wei035 in 14/18; and Wei044 in 13/18. These cultivar amplification failures could be due to polymorphism in primer sequences or the absence of a gSSR locus. A future study comparing gSSR sequences across diverse plant materials could help answer this question. Including more cultivars involved in breeding programs could also help determine if there are other species in the breeding background of the cultivars with gSSR loci that do not amplify consistently. The total number of private alleles per cultivar at the locus level ranged from zero to six (Table 2). The three cultivars with the largest numbers of private alleles were ‘Pink Poppet’ (six), ‘Suzanne’ (five), and Towers of Flowers^®^ Apple Blossom (four).

**Figure 2:**
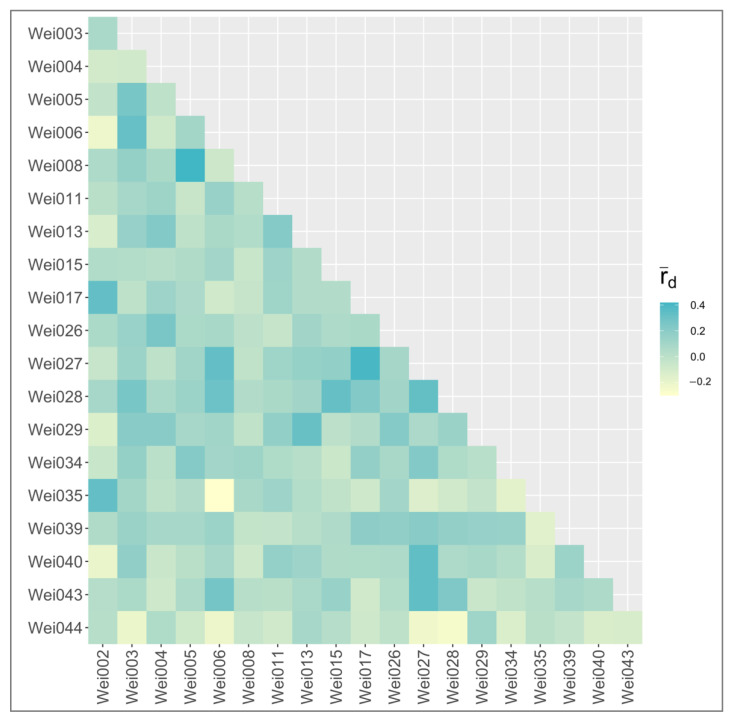
Pairwise linkage disequilibrium of all 20 genomic simple sequence repeat (gSSR) markers developed for *Weigela* cultivars. Pairwise ${\bar{r}}_{d}$ was calculated for each locus pair and visualized with a heatmap to infer any associations between loci. The darker the square, the more of an association there is between the pair of loci and vice versa.

Pairwise linkage disequilibrium was estimated between each pair of markers using the standardized index of association (${\bar{r}}_{d}$), which accounts for the number of loci used [30]. The maximum ${\bar{r}}_{d}$ value is 1 and within our dataset ${\bar{r}}_{d}$ ranged from −0.30 to 0.42. The largest ${\bar{r}}_{d}$ value, 0.42, was between locus Wei005 and locus Wei008 (Figure 2). The consistently low values of ${\bar{r}}_{d}$ suggest that the selected loci are not in linkage disequilibrium and, therefore, likely are well-dispersed throughout the genome. A published reference genome is not available to confirm the distribution of the markers throughout the genome. The 20 gSSR markers are useful for future population genetics studies and other applications where diversity measurements require the independent inheritance of targeted loci.

### 2.3. Applications with Weigela Cultivars

**Figure 3:**
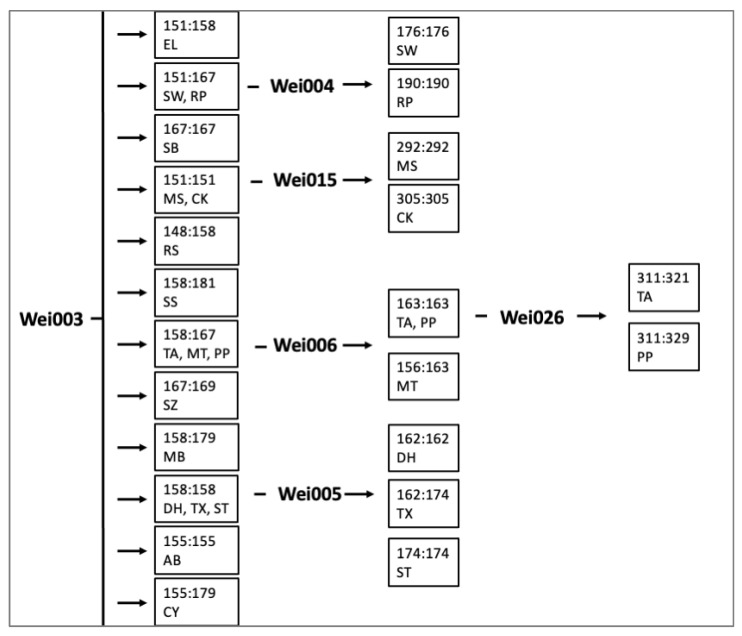
Diagrammatic key to the 18 *Weigela* cultivars included in this study based on allele size. AB = Towers of Flowers^®^ Apple Blossom; CK = Crimson Kisses^®^; CY = Towers of Flowers^®^ Cherry; DH = Dark Horse; EL = Electric Love^®^; MB = Minor Black; MS = Date Night™ Maroon Swoon^®^; MT = Minuet; PP = Pink Poppet; RP = Red Prince; RS = Rainbow Sensation™; SB = Sonic Bloom^®^ Pink; SS = Shining Sensation™; ST = Stunner™; SW = Spilled Wine^®^; SZ = Suzanne; TA = Tango; TX = Tuxedo™.

The potential uses of these markers extend beyond future population genetics studies involving *Weigela* as they could also be used to distinguish cultivars. The 18 cultivars utilized in this study can be differentiated with as few as six gSSR markers and three or fewer markers per cultivar (Figure 3). Of the 20 gSSR markers, Wei003 had the highest discriminatory power and harbored eight alleles in the *Weigela* collection (Table 1). 

The parentage of cultivars included in this study was tracked using information available in patents and publications. Breeding information was not available for all cultivars and information found in patents did not always align with the information provided by nurseries. Despite these knowledge gaps and inconsistencies, three groups were identified in our *Weigela* collection based on common parentage (Table 2). Group One is ‘Minuet’ [31] and its descendant lines ‘Tango’ [32], Electric Love^®^ [33], and Stunner™ [34]. Group Two includes ‘Dark Horse’ [35], Tuxedo™ [36], and Towers of Flowers^®^ Cherry [37]. Group Three is ‘Red Prince’ [38] and its descendant lines Crimson Kisses^®^ [39] and Date Night™ Maroon Swoon^®^ [40]. 

**Figure 4:**
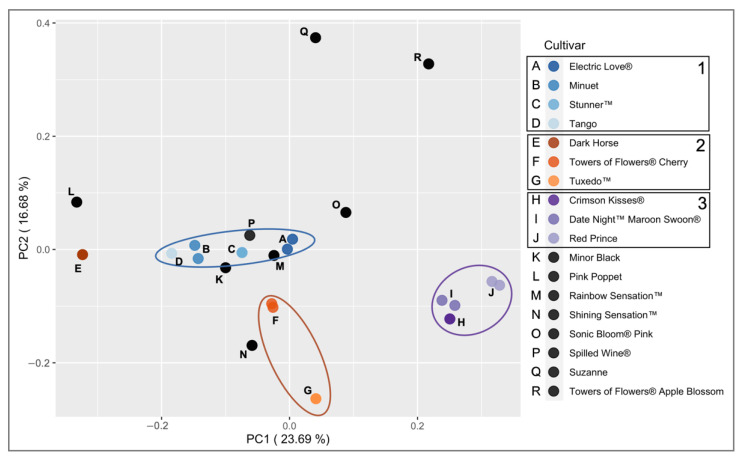
Principal Coordinate Analysis (PCoA) across 18 *Weigela* cultivars using Bruvo’s distance. Percent variance explained for each principal coordinate is listed in the axis label. All cultivars are labeled with letters. Cultivars within identified breeding groups are represented by colored dots. Cultivars in Group One are denoted by shades of blue; Group Two shades of orange; and Group Three shades of purple. Individuals with no deducible breeding group are denoted by black dots. Note individual cultivar samples that are different (i.e., ones where two circles are visible) only vary in the amount of missing data, not alleles—all allele sizes were within four bp of each other, so they were manually corrected before being binned into statistical allelic classes.

**Figure 5:**
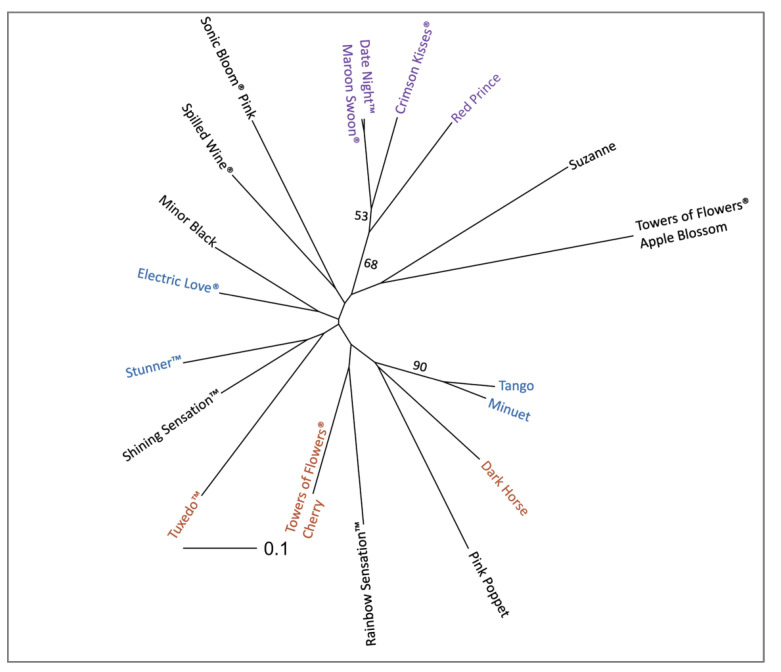
Unrooted neighbor joining tree based on Bruvo’s distance and the BIONJ algorithm across 18 *Weigela* cultivars using 1000 bootstrap replicates. Internal branches with bootstrap values greater than 50 are labeled. Terminal branches representing the two individual cultivar samples all had bootstrap values ≥99. Cultivars within identified breeding groups are represented by a colored font. Cultivars in Group One are denoted by blue; Group Two orange; and Group Three purple. Individuals with no deducible breeding group are denoted by a black font.

Pairwise estimates of Bruvo’s distance were used to create a Principal Coordinate Analysis (PCoA) plot and a neighbor joining tree. Despite not all breeding relationships being apparent using this distance, the first two principal coordinates explained a high percentage (40%) of the variance (Figure 4). ‘Dark Horse’ did not cluster close to the other cultivars in Group Two using this approach. The majority of the other cultivars in the breeding groups are only weakly clustered together, which possibly could be improved if additional cultivars or markers were included, especially parent cultivars such as ‘Victoria’ or ‘Alexandra.’ However, two groups of cultivars consistently clustered together: ‘Minuet’ and ‘Tango’ in Group One and ‘Red Prince’, Date Night™ Maroon Swoon^®^, and Crimson Kisses^®^ in Group Three. These two subsets of the breeding groups were also the only clusters that had bootstrap values greater than 50 when the distance tree was constructed using the BIONJ algorithm (Figure 5). ‘Minuet’ is a parent of ‘Tango’, and ‘Red Prince’ is a parent of both Date Night™ Maroon Swoon^®^, and Crimson Kisses^®^, explaining these close relationships. Group Two’s Tuxedo™ and Towers of Flowers^®^ Cherry grouped together in both PCoA and unrooted neighbor joining tree, matching breeding records. ‘Dark Horse’ was not clustered with breeding Group Two, possibly indicating a more complicated breeding history or misidentification. Future projects that include more cultivars related to Group Two are needed to further disentangle the relationships.

The large number of private alleles identified in our cultivars and high average number of alleles identified with each marker are likely a reflection of the diverse breeding background. Although many *Weigela* species are able to hybridize, *W. florida* and *W. praecox* have been the most important in *Weigela* breeding programs [1]. *Weigela* species have been hybridized extensively, making classification of species and cultivars difficult [1]. Furthermore, it is possible the plants introduced to Europe in the mid-nineteenth-century were actually derived from hybrid crosses among *Weigela* from gardens in East Asia [5]. Overall, the PCoA and neighbor joining tree are not the best method to disentangle the hybridization among species. However, these diverse cultivars can be identified with only six of our gSSR markers, which could be helpful in future breeding efforts. The high cross-amplification frequencies of the gSSR markers across the cultivars from different breeding backgrounds are promising for analyses of other *Weigela* germplasm and species.

## 3. Materials and Methods

### 3.1. Plant Material and DNA Extraction

A total of thirty-four samples were collected from 18 different cultivars of *Weigela* purchased from commercial nursery breeders (Table 2). Leaves were collected from two individual plants for all cultivars except Spilled Wine^®^ and ‘Tango’, which only included one plant. The samples were homogenized using a BeadMill 24 (Thermo Fisher Scientific, Waltham, MA, U.S.) three times at settings of S (speed) = 6.0 m/s, T (time) = 30 s. Samples were frozen in liquid nitrogen between each homogenization. DNA was extracted using the Omega Plant DS kit (Omega Bio-tek, Inc., Norcross, GA, U.S.) following the manufacturer’s protocol, except for heating the elution buffer to 65 °C before use and eluting with 50 µL of the buffer twice, for a final extraction volume of 100 µL. DNA was quantified using a NanoDrop Lite Spectrophotometer (Thermo Fisher Scientific, Waltham, MA, U.S.).

### 3.2. DNA Sequencing and Primer Design

DNA was extracted from leaves of *Weigela* Spilled Wine^®^ collected from Pope’s Creekside Nursery, Knoxville, TN, U.S. in 2021. Extracted DNA was used as a template for sequencing with Illumina MiSeq version 3, 600-cycle (2 × 600) kit (Illumina) at the University of Tennessee Next-Gen Illumina Sequencing Core Facility, Knoxville, TN, U.S. The paired-end, MiSeq raw reads are available at NCBI Bioproject PRJNA819382.

Quality assessment of the reads was done with FastQC v0.11.7 [41] before and after trimming and quality filtering with Trimmomatic v0.39 (minimum read length of 36, minimum quality of 30) [42]. The quality-filtered reads were then assembled into unitigs with ABySS version 2.1.4 [43]. DustMasker v1.0.0 [44] was then used to mask low complexity regions of the genome. Both masked and unmasked FASTA files were used as input files for a custom Perl script [45] that identifies SSRs within sequences and designs primers with Primer3 version 2.5.0 (amplicon range of 150 to 500 bp) [46]. The data were filtered to include only gSSR sequences with 8 to 30 dinucleotide repeats, 7 to 20 trinucleotide repeats, or 6 to 15 tetranucleotide repeats.

### 3.3. Primer Screening and Optimization

A total of fifty primers were selected based on the repeat motif and expected size to allow multiplexing in the future. There were 26 di-, 14 tri-, and 10 tetra-motifs, all with amplicon sizes ranging from 150 to 425 bp which were randomly selected at 25 bp intervals to test with PCR. All 50 primers were analyzed on the 34 samples in 10 µL PCR reactions containing 2 µL of genomic DNA (2 ng/µL), 5 µL 2X Accustart II PCR Supermix (QuantBio, Beverly, MA, U.S.), and 1 µL (2.5 µM) of each forward and reverse primer. Amplifications were performed in 96-well plates using the following thermal profile: 94 °C for 3 min, 15 cycles of touchdown [47]: 94 °C for 40 s, 63 °C −0.6 °C/cycle for 15 s, 72 °C for 30 s, and 20 cycles of 94 °C for 40 s, 57 °C for 40 s, 72 °C for 30 s, with a final extension of 72 °C for 4 min. Amplified PCR fragments were visualized using a QIAxcel Advanced Capillary Electrophoresis system (Qiagen, Germantown, MD, U.S.), aligned using an internal 15/600 bp alignment marker (Qiagen), and length determined using the 25 to 500 bp size marker (Qiagen). Fragments were analyzed using ScreenGel program version 1.6.0 (Qiagen). Loci that failed to amplify or resulted in spurious bands in more than 30% of the samples were discarded from the study. All samples that failed to amplify were subjected to two additional attempts before being scored as missing data. Due to cultivars being clonally propagated and the resolution of the QIAxcel Advanced Capillary Electrophoresis system, alleles that were within four bp of each other were manually corrected to have the same allele size before binning the alleles into statistical allelic categories with FlexiBin [48].

### 3.4. gSSR Data Analysis

All data analyses were conducted with R version 4.1.2 [49], and code is available on github. The number of alleles, percent missing data, and private alleles were calculated with *poppr* version 2.9.3 [50]. *Poppr* was also used to estimate pairwise linkage disequilibrium using the standardized index of association (${\bar{r}}_{d}$). Bruvo’s distance matrix was also calculated with *poppr*. Principal coordinate analysis (PCoA) was performed with *ape* version 5.5 [51]. The first two principal coordinates were visualized with *ggplot2* version 3.3.5 [52]. Cailliez’s correction was used to transform data with *ade4* version 1.7–18 [53]. The untransformed and transformed data were used to determine which algorithm resulted in the highest correlation coefficient. Tested algorithms included FastME balanced, FastME OLS, unweighted pair group method with arithmetic mean (UPGMA), neighbor joining, and BIONJ. All functions for these algorithms are available in *ape*. BIONJ had the highest correlation coefficient and was used to create a genetic distance tree with 1000 bootstrap replicates with *poppr.* The distance tree was visualized with *ape* [51].

## 4. Conclusions

To our knowledge, this is the first study developing genetic markers for *Weigela* cultivars from breeding programs primarily centered around *W. florida*. These resulting 20 gSSR markers can be used in future studies for identifying cultivars, improving breeding programs, and assessing genetic diversity and population structure. These markers appear to be in linkage equilibrium and are highly variable across *Weigela* cultivars. The consistent cross-amplification of the gSSR markers among cultivars from various breeding programs suggests these markers will also be informative in the broader *Weigela* germplasm and related taxa.

## Figures and Tables

**Table 1:** Characteristics of the 20 genomic SSRs (gSSRs) developed for Weigela cultivars.

Locus	GenBank Accession	Primer Sequences	Repeat Motif	Allele Size Range (bp)	Number of Alleles	Missing Data (%)
**Wei002**	OM158066	F: ACATCATCATCACTTGGGTGG	[GAAA]_6_	135–150	5	17.65
		R: ACCAGCACTTTCAATCTTCC				
**Wei003**	OM158067	F: ACATTCCAAGGCCACAAATGC	[TC]_9_	148–181	8	0
		R: GTGGTCCTTGAATGTGTTTCATACG				
**Wei004**	OM158068	F: TGCACCTCAAATGAGACACC	[CT]_8_	150–202	6	0
		R: AGGAGGTGGAGGAGACAAGG				
**Wei005**	OM158069	F: TCGCTGATCGTTTGGAGTCC	[TCT]_11_	159–192	5	0
		R: AGCAAAGTAACCCTAGACAGG				
**Wei006**	OM158070	F: TCCTTCAATGGAGAGAGCCC	[ATAG]_8_	156–175	4	0
		R: CAGAACTTGAATTCATGTTGTTGCC				
**Wei008**	OM158071	F: GCGTGACAAATTGCTTACTTGG	[TATC]_8_	174–201	6	11.76
		R: ACATGGTTCAACAGTCTCCC				
**Wei011**	OM158072	F: TTTCTCAGCAACCAAACCGC	[ACAT]_6_	237–249	4	0
		R: AAAGCACAAGCACAGAAGGG				
**Wei013**	OM158073	F: CCTCAAGAAGAAAGCGCTGC	[GAA]_10_	280–297	5	0
		R: ACCAGAGAAATGTGTAACGC				
**Wei015**	OM158074	F: GAGTGCCAATAGCCAAACCC	[AT]_9_	292–325	6	0
		R: TCGAAAGGTGGACCAACTGG				
**Wei017**	OM158075	F: TCTTCTCATTTGGTGTAGCG	[AAT]_11_	201–217	4	5.88
		R: CGCCACCAATTTGGGTAACG				
**Wei026**	OM158076	F: GTGATTGAGTTTGAGCCGCC	[TA]_13_	304–329	6	0
		R: CATGCACCACACCTTCATGC				
**Wei027**	OM158077	F: GTAAACCAATCAGGCGCACC	[CAT]_7_	340–362	7	0
		R: GCACAAGCAAAGAAGCCGG				
**Wei028**	OM158078	F: GAACCACAACTCAAGCTCCG	[TATG]_7_	308–340	6	0
		R: TCCGACGATTATGCTCACCG				
**Wei029**	OM158079	F: CAATACGAGAAGTGACGCTACC	[TC]_10_	348–361	6	0
		R: CCCTTGCATTAAGAGGGTGC				
**Wei034**	OM158080	F: TCATGCAGTCTAAGCCCACC	[CATA]_6_	383–432	6	0
		R: GGTTACCGCGTGAAGTATGC				
**Wei035**	OM158081	F: TCCACTTCAACCTGAGCTGC	[AG]_8_	388–404	7	23.53
		R: TTAGTGACCACATCGTGACG				
**Wei039**	OM158082	F: TTTCTGCCTAACCAAATCAGCC	[AG]_9_	149–197	7	8.82
		R: CTCACACGTACCACTCTAGCC				
**Wei040**	OM158083	F: TTCAGTTGAAAGACCAACCG	[GA]_8_	171–221	5	0
		R: CACATTGAGAGAGCAATAATTTCCC				
**Wei043**	OM158084	F: AAGAGTTCCGTCCATGCTGG	[AG]_9_	266–286	6	0
		R: GCGAACAAGCTCAATTCCGG				
**Wei044**	OM158085	F: TCTAAAGCTCCATGGTCGGC	[AC]_10_	299–301	2	29.41
		R: AGTGCCAATAGCCAATACCC				

**Table 2:** *Weigela* cultivars used in this study, including breeding information and private alleles detected with 20 genomic SSRs (gSSRs).

Group ^a^	Taxa	Nursery ^b^	US Patent Number/Publication	Breeding Information ^c^	Private Alleles (n)
1	*W.* ‘ZR1’ Electric Love^®^	Pope’s Creekside Nursery	USPP30065	*W.* ‘Tango’ × Open pollinated	0
1	*W.* ‘Minuet’	Nature Hills	Svejda, 1982 ^d^	*W.* ‘Purpurea’ × *W.* ‘Dropmore Pink’	2
1	*W.* ‘Spring 2’ Stunner™	Wayside Gardens	USPP30185	Open Pollinated *W.* ‘Tango’	0
1	*W.* ‘Tango’	Pixie Gardens	Svejda, 1988 ^e^	*W.* ‘Minuet’ × *W.* ‘Nana variegata’	1
2	*W.* ‘Dark Horse’	Nature Hills	USPP14381	*W.* ‘Victoria’ ^g^ × *W.* ‘Foliis Purpureis’	1
2	*W.* ‘TMWG16-04’ Towers of Flowers^®^ Cherry	Wayside Gardens	USPP31915	Open pollination *W. florida* ‘WG13003’ × *W. florida* ‘Alexandra’? ^h^	1
2	*W.* ‘Velda’ Tuxedo™	Wayside Gardens	USPP26842	*W.* ‘Milk and Honey’ × *W. florida* ‘Alexandra’	3
3	*W.* ‘Slingco 1’ Crimson Kisses^®^	Arbor Foundation	USPP23654	*W.* ‘Evita’ × *W.* ‘Red Prince’	1
3	*W.* ‘Slingco 2’ Date Night™ Maroon Swoon^®^	Pope’s Creekside Nursery	USPP26841	Open pollinated *W.* ‘Red Prince’	2
3	*W.* ‘Red Prince’	Nature Hills	Weigle, 1991 ^f^	*W.* ‘Vanicek’ × *W.* ISU41	3
N/A	*W.* ‘Verweig 3’ Minor Black	Wayside Gardens	N/A	unknown	0
N/A	*W.* ‘Pink Poppet’	Nature Hills	US 20030033647 P1	*W. florida* ‘Venusta’ × *W. hybrida* ‘Eva Rathke’	6
N/A	*W.* ‘Kolmagira’ Rainbow Sensation™	Nature Hills	USPP20384	Cross-pollination of unnamed seedling	2
N/A	*W.* ‘Bokrashine’ Shining Sensation™	Nature Hills	N/A	unknown	2
N/A	*W.* ‘Bokrasopin’ Sonic Bloom^®^ Pink	Proven Winners	USPP24572	Open Pollinated *W. hybrida* 93118	1
N/A	*W.* ‘Bokraspiwi’ Spilled Wine^®^	Pope’s Creekside Nursery	USPP23781	Open Pollinated *W. hybrida* 93115	2
N/A	*W.* ‘Suzanne’	Nature Hills	N/A	unknown	5
N/A	*W.* ‘TMWG16-02’ Towers of Flowers^®^ Apple Blossom	Wayside Gardens	USPP32237	Open Pollinated *W. florida* ‘WG13001’	4

^a^ Breeding group the cultivar is a part of; determined by breeding information found in patent or publication. ^b^ Nursery the tested samples were purchased from. ^c^ Breeding information found in patent or publication. ^d^ Svejda, F. (1982). Minuet *Weigela*. *Can. J. Plant Sci*. 62, 249–250. ^e^ Svejda, F. (1988). *Weigela* Cultivars Tango and Polka. *HortScience* 23(4), 787–788. ^f^ Weigle, J., and Stephens, L. (1991). ‘Red Prince’ *Weigela*. *HortScience* 26(2), 218–219. ^g^ ‘Victoria’ is a parent of ‘Alexandra’. ^h^ Possible male parent.

## Data Availability

The data presented in this study are openly available at NCBI Bioproject PRJNA819382. R code can be found on github https://github.com/NowickiLab/Weigela_TrinityPhamm/blob/main/weigela_code_final.R (accessed on 28 May 2022).

## Supplementary Materials

The following supporting information can be downloaded at: https://www.mdpi.com/article/10.3390/plants11111444/s1, Table S1: Allele sizes for all 34 samples at 20 loci.
